# Delivering Telerehabilitation to COVID-19 Inpatients:A Retrospective Chart Review Suggests It Is a Viable Option

**DOI:** 10.1007/s11420-020-09774-4

**Published:** 2020-07-16

**Authors:** Kelsey Rosen, Monika Patel, Cecelia Lawrence, Brianne Mooney

**Affiliations:** Rehabilitation Department, Hospital for Special Surgery, 535 E 70th Street, New York, NY 10021 USA

**Keywords:** coronavirus, COVID-19, telehealth, telerehabilitation, physical therapy, inpatient, rehabilitation

## Abstract

**Background:**

Guidelines for physical therapy management of patients hospitalized with COVID-19 recommend limiting physical therapists’ contact with patients when possible. Telehealth has been viewed as “electronic personal protective equipment” during the COVID-19 pandemic; although telerehabilitation has been shown to be effective with outpatients, it is unknown whether it is a viable option for hospitalized patients.

**Purpose:**

Our facility developed an algorithm for the use of a physical therapy telerehabilitation program for inpatients with COVID-19. We sought to investigate the safety and viability of the program.

**Methods:**

We conducted a retrospective chart review of patients admitted with a diagnosis of COVID-19 who received either telerehabilitation only or a combination of telerehabilitation and in-person rehabilitation. Based on the algorithm, COVID-19 inpatients were selected to receive telerehabilitation if they could ambulate independently, could use technology, had stable vital signs, required minimal supplemental oxygen, and were cognitively intact. We analyzed data of inpatients who received telerehabilitation only, which included patient education, therapeutic exercises, and breathing techniques.

**Results:**

Of 33 COVID-19 inpatients who received telerehabilitation, in-person rehabilitation, or a combination of the two, 12 patients received telerehabilitation only (age range, 33 to 65 years; all but one male). They demonstrated independence with their individualized home exercise programs in one to two sessions, did not require an in-person rehabilitation consultation, did not require increased oxygen, experienced no exacerbation of symptoms, and were discharged home.

**Conclusions:**

Inpatient telerehabilitation appears to be a viable option for selected hospitalized patients with COVID-19 and may be a safe way of delivering inpatient rehabilitation to isolated or at-risk populations. At our hospital, the use of inpatient telerehabilitation reduced staff exposure while providing important education and services to patients. To our knowledge, no studies have investigated the use of telerehabilitation for hospitalized patients, including those with COVID-19. Our findings suggest that this innovative approach warrants further study.

**Electronic supplementary material:**

The online version of this article (10.1007/s11420-020-09774-4) contains supplementary material, which is available to authorized users.

## Introduction

In late March of 2020, Hospital for Special Surgery (HSS), an orthopedic surgical hospital in New York City, began admission of patients with COVID-19 to reduce the strain on neighboring hospitals that were over capacity. Due to the contagious nature of the novel coronavirus, SARS-CoV-2, guidelines based on expert consensus recommended limiting direct contact between rehabilitation therapists and patients with COVID-19 [23]. The guidelines proposed the use of telehealth options when possible for screening and potentially treating patients [5, 23]. Telemedicine can be used as electronic personal protective equipment (PPE) by decreasing risk of exposure and contamination to both patient and practitioner [16].

Telerehabilitation is defined as the “delivery of rehabilitation and habilitation services via information and communication technologies. . . . Telerehabilitation services can include evaluation, assessment, monitoring, prevention, intervention, supervision, education, consultation, and coaching” [21]. Physical therapists provide care to the COVID-19 population to prevent functional decline and manage physical limitations [20, 23]. Patients admitted with COVID-19 commonly present with fever, dyspnea, cough, hypoxia, and fatigue, which may limit their overall functional ability [6, 22]. A clear need for telehealth services emerged to provide rehabilitation while also protecting staff; thus, an inpatient telerehabilitation program was established at HSS.

We found no published studies on the effects of inpatient telerehabilitation services in this novel population, although telerehabilitation has been shown to be effective in outpatient settings [1, 9, 11]. In July 2018, HSS implemented a telerehabilitation program, HSS@Home, as an alternative means of providing home-based physical therapy (PT), primarily to selected patients after total hip or total knee arthroplasty [11]. We surmised that with some logistical modifications, telerehabilitation could be used to treat an inpatient population. The established technology used for HSS@Home was modified for the inpatient setting for patients with COVID-19.

The purpose of the inpatient telerehabilitation PT program for COVID-19 patients was to safely assess barriers to discharge, deliver patient education, and provide a home exercise program (HEP) all while minimizing staff exposure. Criteria for success of the program were determined by the ability to create an algorithm, successfully train staff, deliver education efficiently, maintain patient and staff safety during intervention, and ensure patients were able to discharge home safely.

The objectives of this retrospective chart review were to ascertain how the inpatient telerehabilitation program was implemented and whether it was a viable option for COVID-19 patients.

## Methods

This retrospective chart review was approved by the Institutional Review Board at HSS. Data were retrospectively collected from electronic medical records of all inpatients with COVID-19 who received telerehabilitation services between April 8 and May 12, 2020. We further focused our data gathering on inpatients who had telerehabilitation PT only.

### COVID-19 Rehabilitation Response Algorithm

**Fig. 1:**
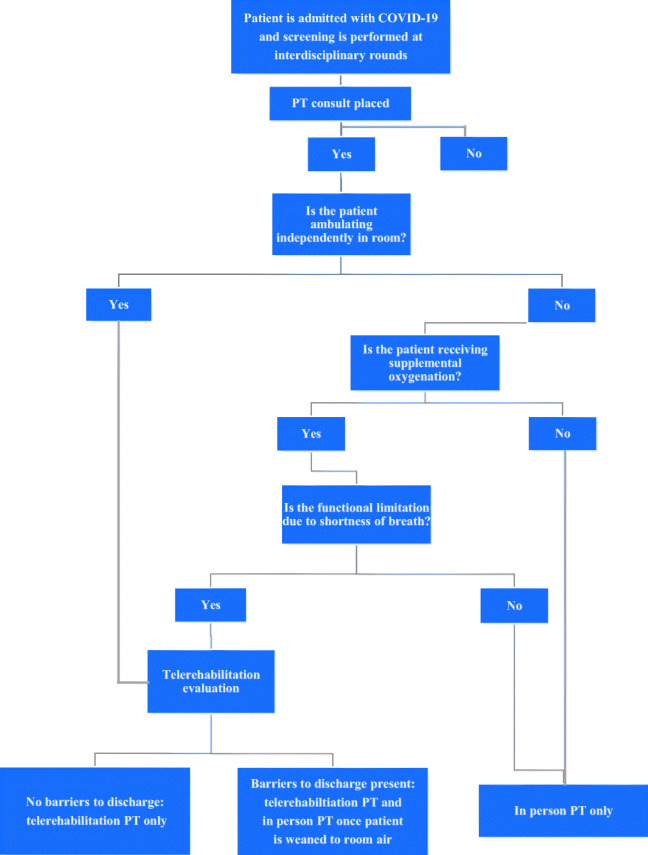
COVID-19 rehabilitation response algorithm. Barriers to discharge home included but were not limited to endurance impairment identified in order to perform stair negotiation, ability to self-isolate from family members, and/or need for an assistive device for ambulation secondary to identified functional impairments.

All patients admitted to HSS with COVID-19 were screened for PT consults through interdisciplinary rounds via a conference call. The interdisciplinary team consisted of an attending physician or a physician assistant, a case manager, a nurse manager, and a physical therapist. To determine which type of PT services the patients required, a COVID-19 rehabilitation response algorithm (Fig. 1) was created based on international expert consensus guidelines [23]. The guidelines recommend varying levels of PT intervention, depending on disease severity and symptomatology. Six therapists were trained and independent with the technology after a 60-min training session. Most patients (10 of 12) required only one telerehabilitation session to demonstrate independence with their individualized HEP and did not require any in-person PT. Using the algorithm, three groups were identified: (1) patients who required in-person PT only, (2) patients who required telerehabilitation PT only, and (3) patients who required a combination of in-person and telerehabilitation PT.

The primary factor in determining whether a patient required in-person PT was functional mobility status. In-person PT was provided to patients with functional mobility impairments who were not limited by shortness of breath. Telerehabilitation PT only was provided to patients who were mobilizing independently, either on or off supplemental oxygen, or to patients whose mobility was limited by shortness of breath but who did not have any barriers to discharge. The combination of in-person PT and telerehabilitation was provided when functional mobility limitations due to shortness of breath and barriers to discharge home were present. In these instances, telerehabilitation was performed first for education, therapeutic exercises, and assessment of barriers to discharge. Once the patient was weaned from supplemental oxygen, in-person PT addressed the barriers to discharge and assessed any residual functional limitations.

In order to qualify for telerehabilitation PT only, COVID-19 inpatients had to be able to ambulate independently in their rooms with a nurse and needed PT education, therapeutic exercises, and/or breathing techniques. They also had to demonstrate the ability to use technology, have stable vital signs (heart rate, blood pressure, oxygen saturation), require minimal supplemental oxygen (2 L/min or less), and be cognitively intact. The presence of functional deficits, a discharge destination of acute or subacute rehabilitation, and/or known physical barriers to discharge excluded patients from the telerehabilitation only program.

### Staff Training

**Fig. 2:**
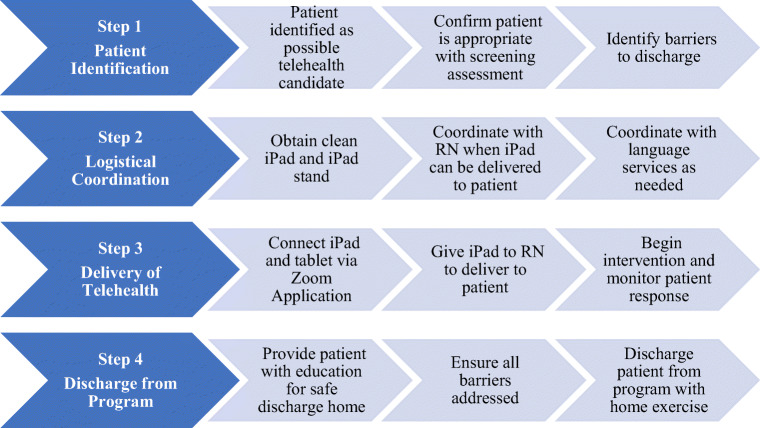
Telerehabilitation program implementation. If patients required or benefitted from more than one session, continued assessment of appropriateness was performed through steps above.

Physical therapists volunteered to treat patients with COVID-19 and were assigned to specific teams (critical care/intensive care unit, general medical COVID floors, and telerehabilitation) based upon clinical experience and staffing needs. The therapists assigned to inpatient telerehabilitation PT were trained to use Zoom for video communications (Zoom Video Communications, San Jose, CA, USA). Training was performed by the informatics team and took approximately 1 h (Table 1). Administration of this program required identification of appropriate patients, logistical coordination, delivery of telerehabilitation, and discharge of patients from the program (Fig. 2). In an effort to minimize staff exposure to COVID-19, physical therapists scheduled telerehabilitation sessions and delivery of iPads and written material in conjunction with nursing care. Once schedules were determined, physical therapists contacted Language Services for coordination of interpreters as needed.

**Table 1:** Telerehabilitation resources

Tablets/chargers/headsets for therapist use	
iPads and iPad stands for patient use	
Notepads for documentation	
A binder with physical therapist communication sheet and patient exercises for easy distribution in multiple languages	
Personal protective equipment needed to enter room in case of an emergency*	

*During a telehealth session, each therapist was prepared by wearing goggles, an N95 mask, and a surgical mask in case it would be necessary to enter the patient’s room in an emergency

The telerehabilitation physical therapists were stationed near the external telemetry and oxygen saturation (SpO_2_) monitors for immediate vital sign feedback during sessions. Treatment was adjusted based on peripheral oxygen saturation, heart rate, respiratory rate, and blood pressure during sessions. The following interventions were performed via telerehabilitation, and supplemental written material was provided (Online Resource 1).

### Patient Education

Physical therapists educated patients on the COVID-19 disease process and on energy conservation techniques such as activity pacing [10, 12]. Due to the cardiopulmonary limitations in the COVID-19 population, the modified Borg scale (MBS) was used to monitor patient response to therapy [4, 14, 15, 17]. If the activity was rated higher than 4 on the MBS, the patient was instructed to modify the activity. Additionally, traditional discharge education on fall prevention and modifications to the home environment for safety was performed.

### Therapeutic Exercise

Patients assessed via telerehabilitation were provided individualized therapeutic exercises based on early anecdotal reports of high fatigability in patients with COVID-19 and clinical knowledge of therapeutic interventions for similar disease processes such as acute respiratory distress syndrome [3, 14, 15, 23]. Based on muscles important to maintain functional mobility such as ambulation and sit-to-stand transfers, lower extremity muscle groups including gluteals and quadriceps were targeted during supine, seated, and standing exercises [2, 24, 25]. Upper extremity exercises were provided to aid in respiratory function, posture, and functional mobility [18].

Typically, patients performed one set of five to ten repetitions of each exercise based on rate of perceived exertion (RPE) with physical therapist supervision via telerehabilitation. Patients were encouraged to complete the same set of exercises an additional two times, without supervision throughout the day, for a total of three sets per day. Patients were taught to progress based on RPE through the use of antigravity motions, resistance bands, and repetitions of exercise (Online Resource 1).

### Breathing Techniques

At our institution, respiratory therapy traditionally performs respiratory inventions. Due to limited availability of respiratory therapists at our institution during COVID-19, physical therapists assumed responsibility for performing select, noninvasive respiratory interventions. The breathing techniques and exercises used in the telerehabilitation program were based on the PT guidelines adapted from expert consensus [23, 26, 27]. Patients with a productive cough were taken through the active cycle of breathing technique (ACBT) and cough etiquette [23], the latter important in this population due to the risk of SARS-CoV-2 transmission by aerosol generation. Pursed-lip breathing was taught to patients who were short of breath as a form of breathing control. Straw breathing was taught as a form of positive expiratory pressure to improve ventilation in select patients [23] (Online Resource 1).

### Discharge Criteria

Patients were discharged from telerehabilitation when they could monitor exertion through the use of the MBS, verbalize contraindications to activity progression, and demonstrate independence with HEP and when physical barriers to return home safely were addressed. Oxygenation parameters for medical clearance were determined by the medical team. The standard parameters at HSS for discharge required patients to maintain SpO_2_ over 94% on room air (RA) at rest and over 90% with ambulation for 24 h prior to discharge. This was monitored by nursing staff.

Based on guidelines for COVID-19 functional mobility progression, patients were educated to maintain a metabolic equivalent for task (MET) level of 3 or lower for the first 6 weeks of recovery at home [14, 15]. Therefore, patients who had a flight of stairs or more to access their home were recommended ambulance transport home.

## Results

Of the 33 inpatients who received telerehabilitation PT services, 12 patients had telerehabilitation PT only. Of those 12 inpatients, 91% were male; median age was 56 years, and common signs and symptoms on admission included cough, shortness of breath, fever, tachycardia, and chest pain (Table 2). Shortness of breath was the most common symptom, seen in 83% of the patients. Upon admission, patients required an average of 3.5 L/min supplemental oxygen, with a range of 2 to 6 L/min, via nasal cannula. At the time of the telerehabilitation consultation, all patients had been weaned to 1.5 L/min or less of supplemental oxygen, and all patients were independently mobile (Table 3).

**Table 2:** Patient demographics

Patient	Age (years)	Gender	Signs and symptoms	PMH/comorbidities
1	42	M	SOB, fever, tachycardia	None
2	60	M	SOB, cough	HTN, pre-DM, HLD, former smoker, back pain, PSA
3	65	M	Fever, cough, fatigue, myalgia	HTN, HLD, DM
4	58	M	Fatigue, tachycardia, headache, midsternal chest pain, constipation	HTN, hypothyroidism, DM, tobacco abuse, HLD, class 1 obesity
5	45	M	SOB, cough, pleuritic right-sided chest pain	DVT, PE, DM
6	46	M	SOB, fever, chest pain	PE, transaminitis
7	54	M	SOB, fever, cough, diarrhea	HTN, DM
8	66	M	SOB, pleuritic chest pain	PNA, depression
9	59	M	SOB, cough	None
10	33	F	SOB, anxiety	PNA, DM2, transaminitis
11	59	M	SOB, tachycardia	HTN, HLD, DM2
12	47	M	SOB, cough, diarrhea, midline tenderness	Hypoxia, bacterial PNA, hepatitis, malnutrition, headaches, dizziness, difficulty sleeping, hyponatremia

*M* male, *F* female, *PMH* past medical history, *SOB* shortness of breath, *PNA* pneumonia, HTN hypertension, *DM* diabetes mellitus, *HLD* hyperlipidemia, *PSA* prostate-specific antigen, *PE* pulmonary embolism, *DVT* deep vein thrombosis

**Table 3:** Patient chart review

Patient^a^	Admission date (2020)	Telerehabilitation evaluation date (2020)	LOS (days)	O_2_ on admission	O_2_ during session	No. sessions	Intervention	Discharge services
1	4/8	4/15	8	6 L/min	RA	1	PtEd, therex, BrT	No services
2	4/9	4/16	11	3 L/min	RA	1	PtEd, therex, BrT	No services
3	4/16	4/18	2	5 L/min	RA	1	Therex	VNS
4	4/17	4/19	9	4 L/min	RA	1	PtEd, therex, BrT	HSS@Home
5	4/20	4/23	11	3 L/min	1.5 L/min RA	2	PtEd, therex, BrT	HSS@Home
6	4/21	4/23	10	2 L/min	0.5 L/min RA	2	PtEd, therex, BrT	VNS then HSS@Home
7	4/15	4/25	11	3 L/min	1 L/min	1	Therex, BrT	HSS@Home
8	4/14	4/25	14	3 L/min	1 L/min	1	PtEd, therex, BrT	HSS@Home
9	4/17	4/25	8	3 L/min	RA	1	PtEd, therex, BrT	HSS@Home
10	4/22	4/26	10	2 L/min	1 L/min	1	PtEd, therex, BrT	HSS@Home
11	4/22	4/26	7	4 L/min	1 L/min	1	PtEd, therex, BrT, coughing	HSS@Home
12	4/19	4/26	8	4 L/min	RA	1	PtEd, therex, BrT, coughing	HSS@Home

*Telerehab* telerehabilitation, *O*_*2*_ oxygen, *L/min* liters/min, *RA* room air, *PtEd* patient education, *therex* therapeutic exercises, *BrT* breathing technique, *VNS* visiting nurse service

^a^All patients had a confirmed diagnosis of COVID-19, had a discharge destination of home, and were discharged on room air

After the telerehabilitation intervention, none of the patients required increased oxygen supplementation or medical care. No adverse events occurred to patients receiving telerehabilitation PT only; therefore, the telerehabilitation therapists were not required to enter patient rooms. The timing of telerehabilitation consultations was dependent on the medical stability of patients. The average patient length of stay was 9.1 days, with telerehabilitation evaluation occurring on average 5 days after admission. At the time of discharge, all 12 patients were discharged without supplemental oxygen and were able to maintain oxygen saturation goals for discharge.

After receiving inpatient telerehabilitation services, 100% of the patient cohort met their PT goals and were discharged home safely when medically appropriate. Of the 12 patients, two were discharged home with no PT services, two were discharged with homecare services, and eight were discharged with an outpatient telehealth program, HSS@Home.

## Discussion

PT practice is changing to meet the urgent needs of patients with COVID-19. Telehealth has been identified as a critically essential service for patients to decrease the spread of COVID-19 and conserve necessary PPE [5]. Inpatient telerehabilitation provided an alternative mode of delivering a valuable service to patients with COVID-19. Success of the inpatient telerehabilitation program was determined based on the ability to create an algorithm, train staff, deliver education efficiently, maintain patient and staff safety during interventions, and ensure patients discharged home safely.

The limitations of this study include its retrospective and descriptive design, its small sample size, the lack of comparative analysis or control group, and the lack of standardized outcome measures to track functional progress over time or assess patient satisfaction. Due to the rapid influx of patients with COVID-19 and the urgency with which this program was created, outcome measures were not collected on all patients consistently; outcome measure assessment was incorporated approximately halfway through the program. We noted a ceiling effect to the interventions performed for some patients due to the inability of the physical therapists to progress patients through therapeutic exercises. While supine and seated therapeutic exercises were successful in targeting lower extremity muscle groups, standing therapeutic exercises were not performed in order to maintain patient safety and prevent adverse events or falls. We expect that some of these challenges could be mitigated as the program is used over time. Despite these limitations, we believe that this retrospective review provides preliminary data on how to implement an inpatient telerehabilitation program and suggests that selected COVID-19 patients can be discharged successfully after this method of treatment. Further study of long-term outcomes will be necessary.

Our physical therapists created and used an algorithm to identify COVID-19 patients appropriate for inpatient telerehabilitation. The algorithm allowed for consistency between staff members in determining which form of PT would be used: in-person PT, telerehabilitation PT, or a combination. Physical therapists were trained as demonstrated by the ability to learn the technology within 60 min. Patient education was delivered efficiently, as most patients required only one session to achieve independence with their HEP and education. Interventions provided were safe for both patients and practitioners as evidenced by no increase in medical care or adverse events and minimized staff exposure. Additionally, all 12 patients were given valuable education through teletechnology and were safely discharged home. No in-person PT intervention was required during hospitalization.

After treating patients via telerehabilitation PT, it became apparent that specific patients would benefit from ongoing monitoring and PT intervention once discharged home. Our facility subsequently expanded the HSS@Home program from an orthopedic only population to include patients with COVID-19. The use of telemedicine is growing and existing literature describes its success [1, 5, 7–9, 13, 19]. There is, however, a lack of research on the strategic implementation of telerehabilitation within the inpatient setting and in the COVID-19 population due to the novel nature of the disease. Our retrospective review suggests that high-risk inpatients in isolation may benefit from this mode of delivery of PT. This method of treatment could be implemented in other infectious diseases with aerosolized or contact modes of transmission, such as Ebola, tuberculosis, or herpes zoster.

While strictly inpatient telerehabilitation appears to be beneficial in this study’s cohort, it could be expanded for use in combination with in-person PT services in both COVID-19 and non-COVID-19 populations. Telerehabilitation could be used as a way to provide therapy sessions to monitor inpatients who do not require daily therapy or to provide a second therapy session in inpatients requiring isolation. Additionally, in the COVID-19 population, telerehabilitation could potentially be routinely implemented early in hospitalization to facilitate identification of barriers to discharge and determine specific PT needs. This would enable assessment for potential durable medical equipment needs, identification of safety concerns, and screening for potential changes in functional status compared with baseline. Telerehabilitation may also have a role in caregiver education in situations where the caregiver is unable to be physically present.

Future efficient implementation of telerehabilitation programs would benefit from uniform setup and a designated, ergonomically conscious, quiet treatment space with additional monitors of patients during sessions.

This retrospective review suggests that telerehabilitation is a viable option for providing safe and effective inpatient PT to patients with COVID-19 meeting select criteria. The use of inpatient telerehabilitation allowed for a reduction of staff exposure and preservation of PPE while providing necessary education and resources to patients. Such a program may be replicated in other institutions for isolation or at-risk populations. Treatment should be specific to each patient’s needs and address individual barriers to discharge. Further exploration of the effectiveness of inpatient telerehabilitation PT strictly as well as in conjunction with in-person therapy warrants further investigation within the COVID-19 and broader inpatient populations.

## Electronic supplementary material

ESM 1 (PDF 590 kb)

ESM 2 (PDF 1224 kb)

ESM 3 (PDF 1224 kb)

ESM 4 (PDF 1224 kb)
