# Supplementary material for: Delivering Telerehabilitation to COVID-19 Inpatients:A Retrospective Chart Review Suggests It Is a Viable Option
Source: HSS J. 2020 Jul 16;16(Suppl 1):64–70. doi: 10.1007/s11420-020-09774-4 (PMC7364751; doi:10.1007/s11420-020-09774-4)
Supplement: Supplementary file 1 — (PDF 590 kb) [file 11420_2020_9774_MOESM1_ESM.pdf]

Online supplementary content: Rosen et al. Delivering telerehabilitation to COVID-19 inpatients: a retrospective chart review suggests it is a viable option. *HSS Journal*. 2020.
DOI: 10.1007/s11420-020-09774-4

Therapeutic Exercise for Bed

Ankle Pumps

Purpose:
- To help strengthen the muscles in your lower leg and improve circulation

Starting position:
- Lie on your back with your legs straight.

Action:
- Move ankles up and down through your full range of motion.
- Repeat ______ repetitions, ______ sets daily.

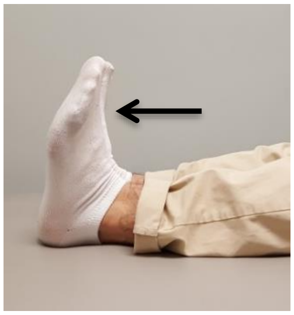
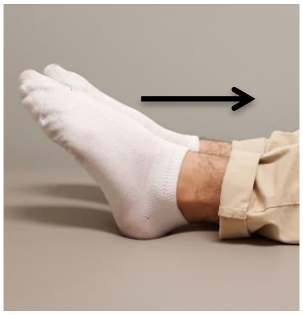

Quadriceps Sets

Purpose:
- To help strengthen the muscles in the front of your thigh

Starting position:
- Lie on your back with your legs straight.

Action:
- Tighten the muscles on the top of your thighs, pressing your knees down into the bed. Hold this contraction for 5 seconds, and then slowly release.
- Repeat ______ repetitions, ______ sets daily.

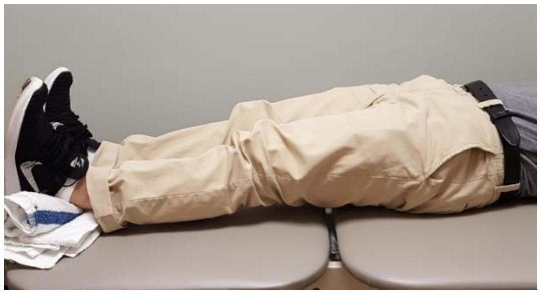

Gluteal Set

Purpose:
- To help strengthen your buttock (gluteal) muscles

Starting position:
- Lie on your back with your legs straight.

Action:
- Gently squeeze your buttocks together. Hold this contraction for 5 seconds, then slowly release.
- Repeat______ repetitions, ______ sets daily.

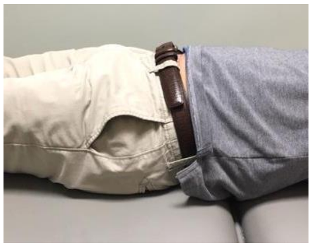
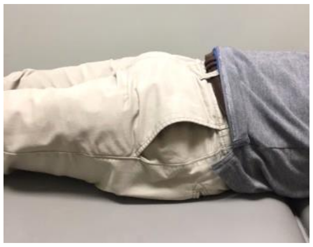

Heel Slides

Purpose:
- To help strengthen muscles that bend your hip

Starting position:
- Lie on your back with your legs straight.

Action:
- Gently slide your heel toward your buttock. Slide it back to the starting position.
- Repeat ______ repetitions ______ sets daily.

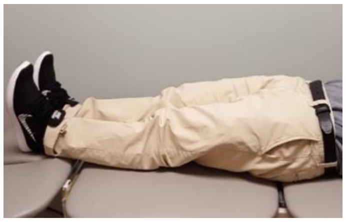
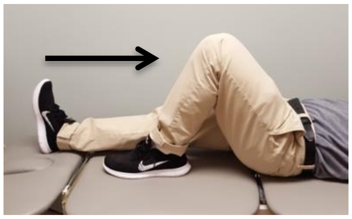

Therapeutic Exercise for Edge of Bed/Chair

Bicep Curl

Purpose:

- To help strengthen your biceps

Starting position:

- Sit on a chair or bed. Place one end of the theraband securely under your foot and grasp the other end in your hand with your palm up towards the ceiling.

Action:

- Keeping your wrist straight, bend your elbow to bring your hand to your shoulder. Slowly return to start position.
- Repeat ___________ repetitions, __________ sets daily.

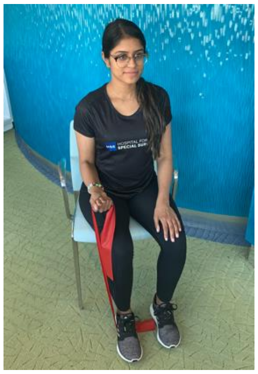

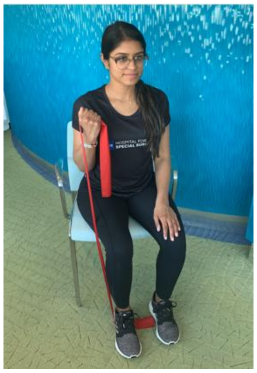

Forward Punches

Purpose:

- To help strengthen your serratus anterior muscle, which helps stabilize the scapula

Starting position:

- Sit on a chair or bed. Begin with band wrapped around your mid back. Grasp the ends of the band in front of you with your elbows bent at your side.

Action:

- Extend your elbows forward and push the band away from your trunk. Hold and slowly return to start position.
- Repeat ___________ repetitions, __________ sets daily.

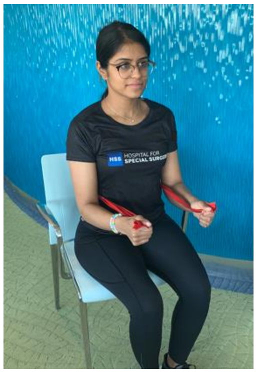

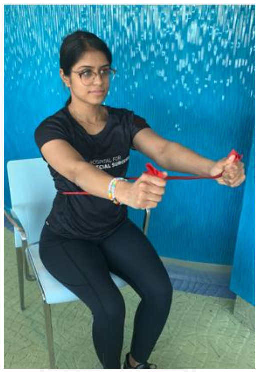

Arm Openers

Purpose:

- To strengthen your upper back and promote improved posture

Starting position:

- Sit on a chair or bed. Hold the band such that your arms are straight out in front of you at shoulder height.

Action:

- Pull the band with both arms and tighten your shoulder blade muscles. Do not lift your shoulders.
- Repeat ___________ repetitions, __________ sets daily.

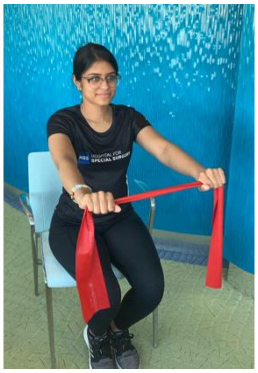

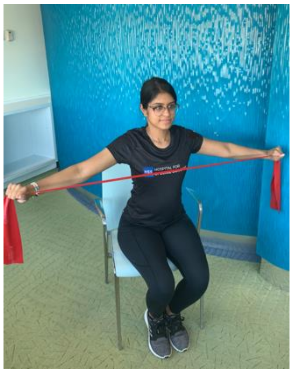

Scapular Retraction

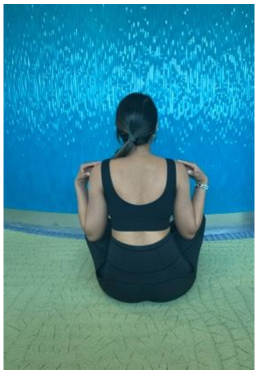

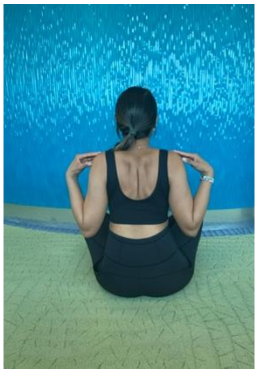

Purpose:
- Strengthen the scapular stabilizers

Starting position:
- Sit on a chair or bed. Place your fingers on your shoulders.

Action:
- Pinch your shoulder blades together without bringing your shoulders up. Hold and slowly return to start position.
- Repeat ____________ repetitions, ___________ sets daily.

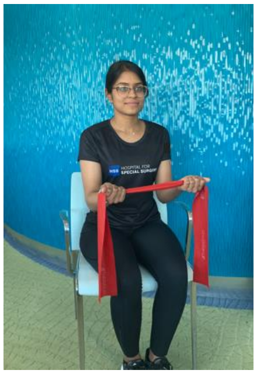

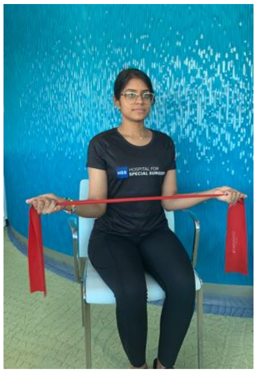

Scapular retraction can be progressed with a band as seen in the picture to the right.

Mini Band Row

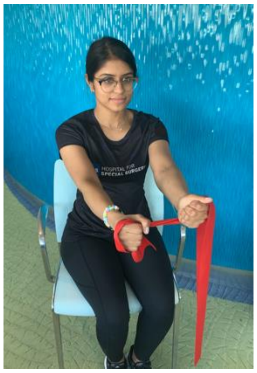

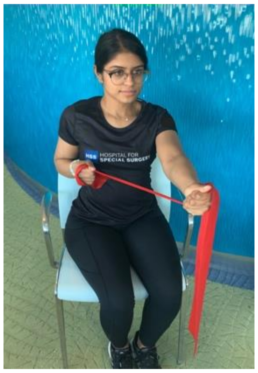

Purpose:
- Strengthen the scapular retractors and back muscles

Starting position:
- Sit on a chair or bed. Hold a band with both hands close to one end. Lift your arms straight forward at chest level with elbows straight.

Starting position:
- Bring one elbow back towards the side of your body while keeping the other arm straight. Keep your pelvis pointing forward. Hold and slowly return to start position.
- Repeat ____________ repetitions, ___________ sets daily.

Seated Knee Straightening

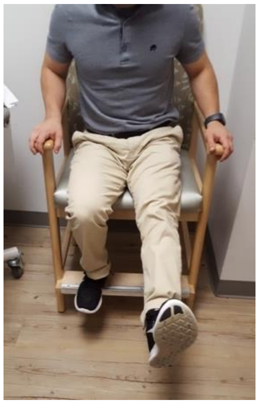
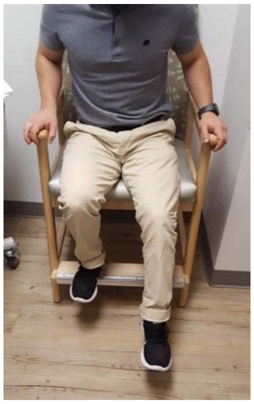

Purpose:
- To help strengthen the muscles on the front of your thigh

Starting position:
- Sit in a chair that is comfortable for you, not too high or low from the ground.

Action:
- Slowly straighten your knee. Return to the resting position.
- Repeat ______ repetitions, ______ sets daily.

Seated Knee Lifts

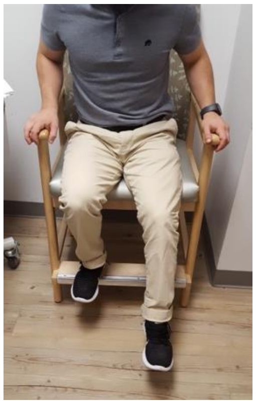
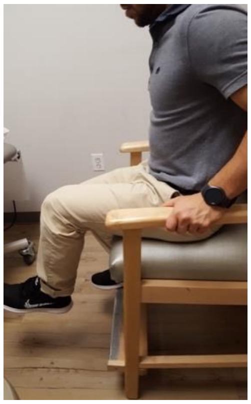

Purpose:
- To help strengthen the muscles that bend your hip

Starting position:
- Sit in a chair that is comfortable for you, not too high or low from the ground.

Action:
- Gently lift your thigh slightly off the chair, keeping you knee bent. Return to the resting position.
- Repeat ______ repetitions, ______ sets daily.

Breathing Activities

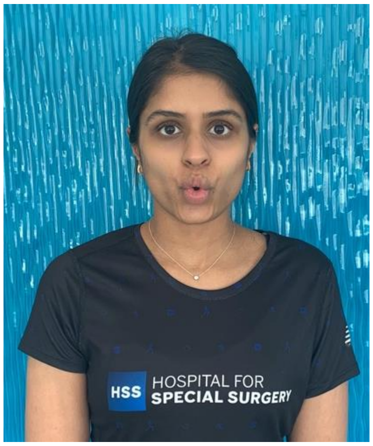

Pursed Lip Breathing

Breathe slowly through your nose for a count of 2 seconds. Blow out gently through pursed lips for a count of 4 seconds.
Repeat 10 times every hour that you are awake.

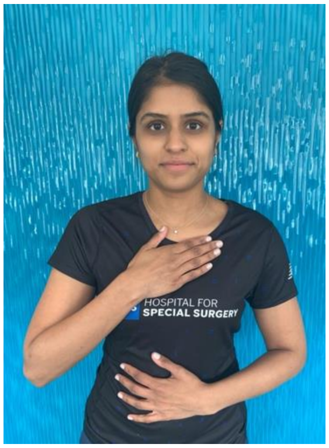

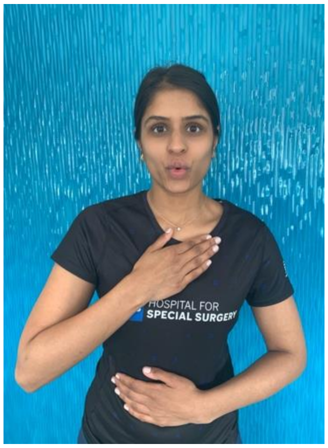

Diaphragmatic Breathing

Lie in bed or sit on a chair with good back support. Place your hands on your belly. Breathe in slowly through your nose and feel your belly expand into your hands. Exhale through pursed lips as your belly flattens.
Repeat 10 times every hour that you are awake.

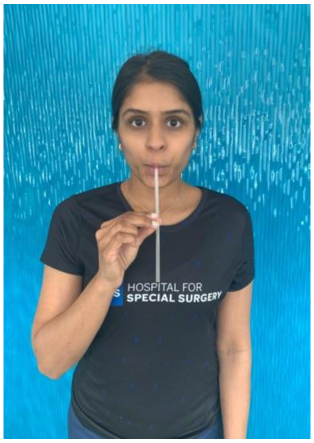

Straw Breathing

Inhale normally and naturally. Exhale fully through a plastic drinking straw, making sure you have exhaled all of the air out of your lungs. Inhale normally (not through the straw). Exhale fully out of the straw.
Repeat 10 times every hour that you are awake.
